# Comparative studies of vertebrate iduronate 2-sulfatase (*IDS*) genes and proteins: evolution of A mammalian X-linked gene

**DOI:** 10.1007/s13205-016-0595-3

**Published:** 2017-04-11

**Authors:** Roger S. Holmes

**Affiliations:** Griffith Institute for Drug Discovery and School of Natural Sciences, Griffith University, Nathan, QLD 4111 Australia

**Keywords:** Vertebrates, Iduronate 2-sulfatase, Amino acid sequence, IDS, X-chromosome, *IDS* gene regulation, Evolution

## Abstract

IDS is responsible for the lysosomal degradation of heparan sulfate and dermatan sulfate and linked to an X-linked lysosomal storage disease, mucopolysaccharidosis 2 (MPS2), resulting in neurological damage and early death. Comparative IDS amino acid sequences and structures and *IDS* gene locations were examined using data from several vertebrate genome projects. Vertebrate IDS sequences shared 60–99% identities with each other. Human IDS showed 47% sequence identity with fruit fly (*Drosophila melanogaster*) IDS. Sequence alignments, key amino acid residues, *N*-glycosylation sites and conserved predicted secondary and tertiary structures were also studied, including signal peptide, propeptide and active site residues. Mammalian *IDS* genes usually contained 9 coding exons. The human *IDS* gene promoter contained a large CpG island (CpG46) and 5 transcription factor binding sites, whereas the 3′-UTR region contained 5 miRNA target sites. These may contribute to *IDS* gene regulation of expression in the brain and other neural tissues of the body. An *IDS* pseudogene (*IDSP1*) was located proximally to the *IDS* gene on the X-chromosome in primate genomes. Phylogenetic analyses examined the relationships and potential evolutionary origins of the vertebrate *IDS* gene. These suggested that *IDS* has originated in an invertebrate ancestral genome and retained throughout vertebrate evolution and conserved on marsupial and eutherian X-chromosomes, with the exception of rat *Ids* on chromosome 8.

**Electronic supplementary material:**

The online version of this article (doi:10.1007/s13205-016-0595-3) contains supplementary material, which is available to authorized users.

## Introduction

Iduronate 2-sulfatase (IDS; EC 3.1.6.13) is responsible for the lysosomal degradation of the glycoaminoglycans, heparan sulfate and dermatan sulfate (Bielicki et al. 1990), and is one of the 19 members of human sulfatase gene families and 17 members of the mouse sulfatase gene families which catalyze the hydrolysis of sulfate esters in the body derived from several catabolic pathways (Ratzka et al. 2010). Many *IDS* gene mutations and IDS deficiencies have been studied in human populations which result in the lysosomal storage of glycoaminoglycans and Hunter syndrome, an X-linked chromosome disease, referred to as mucopolysaccharidosis type 2 (MPS2) (Wilson et al. 1990; Rathmann et al. 1996; Chistiakov et al. 2014; Kosuga et al. 2016). Major clinical features for this rare genetic disease (1:100,000 births) include obstructive and restrictive airway disease, skeletal deformations, cardiac disease, joint contractures and mental retardation (Beck 2011; Tylki-Szymańska 2014; Anekar et al. 2015). Mouse and zebra fish animal models have been used to study the disease in more detail, including studies of *Ids* ^*−*^ */Ids* ^*−*^ knock out mice which have shown that IDS-deficiency generates many of the defects reported for human MPS2 (Garcia et al. 2007). In addition, possible treatments for the disease by enzyme replacement therapy have been investigated (Garcia et al. 2007; Moro et al. 2010; Fusar Poli et al. 2013; Cho et al. 2015; Parini et al. 2015) and a phase I/II clinical trial of intrathecal IDS replacement therapy in children with severe MPS2 has been recently reported (Muenzer et al. 2016).

The gene encoding IDS (*IDS* in primates; *Ids* in rodents) is expressed at high levels in neural tissues, particularly in the cortex, hippocampus, other brain and eye tissues; and is also widely expressed throughout the body (Smith et al. 2014). The enzyme catalyzes the first step in the degradation of glycoaminoglycans, dermatan sulfate and heparan sulfate (Bielicki et al. 1990). Human *IDS* is expressed as three major isoforms which have distinct C-terminal sequences: *IDSa* encoding a 550 amino acid protein, expressed in brain tissues and with a wide tissue distribution; *IDSb*, 460 amino acids also expressed in brain tissues; and *IDSc*, encoding a 446 amino acid enzyme expressed in ductal carcinoma cells and pancreas (Thierry-Mieg and Thierry-Mieg 2006). The genomic organization of the human and mouse *IDS*/*Ids* genes have been reported with 9 exons observed for 24 kb and 22 kbs of DNA, respectively (Wilson et al. 1993; Thierry-Mieg and Thierry-Mieg 2006).

Biochemical and predictive structural studies of human IDS have shown that it comprises several domains: an N-terminus signal peptide (residues 1–25); a propeptide sequence (residues 26–33); five Ca^2+^ binding sites (1 Ca^2+^ per subunit); two active site residues (334Asp and 335His); and seven N-glycosylation sites (Bielicki et al. 1990; Wilson et al. 1990; Kosuga et al. 2016). A predicted tertiary structure has been reported for human IDS (Sáenz et al. 2007), which shows strong similarities with other human sulfatases: GALNS (Rivera-Colón et al. (2012)); ARSA (Chruszcz et al. 2003) and STS (Hernandez-Guzman et al. 2003).

This paper reports the predicted gene structures and amino acid sequences for several vertebrate *IDS* genes and proteins, the predicted structures for vertebrate IDS proteins, a number of potential sites for regulating human *IDS* gene expression and the structural, phylogenetic and evolutionary relationships for these genes and enzymes.

## Methods

### Vertebrate *IDS* gene and protein identification

BLAST studies were undertaken using web tools from NCBI (http://www.ncbi.nlm.nih.gov/) (Camacho et al. 2009). Protein BLAST analyses used human and mouse IDS amino acid sequences previously described (Bielicki et al. 1990; Garcia et al. 2007) (Table 1). Protein sequence databases for several vertebrate genomes were examined using the blastp algorithm (see Holmes 2016). Predicted IDS protein sequences were obtained in each case and subjected to analyses of predicted protein and gene structures.

**Table 1:** Vertebrate IDS Proteins

ID*S* Protein	Species	UNIPROT ID	Amino acids	Subunit MW	pI	*N*-Glycosylation sites	Signal peptide	% Identity human IDS
Human	*Homo sapiens*	P22304	550	61,873	5.2	115, 144, 246, 280, 325, 513, 537	1..25	100
Chimpanzee	*Pan troglodytes*	na	550	61,861	5.2	115, 144, 246, 280, 325, 513, 537	1..25	99
Orangutan	*Pongo abelii*	H2PX10	550	62,083	5.4	115, 144, 246, 280, 325, 513, 537	1..25	96
Baboon	*Papio anubis*	na	550	61,885	5.1	115, 144, 246, 280, 325, 513, 537	1..25	96
Marmoset	*Callithrix jacchus*	F7EJG2	550	61,812	5.4	115, 144, 246, 280, 325, 513, 537	1..25	94
Mouse	*Mus musculus*	Q08890	552	62,186	5.5	117, 146, 248, 282, 515, 539	1..29	86
Rat	*Rattus norvegicus*	Q32KJ4	543	62,370	5.5	117, 146, 248, 181, 515, 539	1..20	85
Cow	*Bos taurus*	F1N2D5	547	61,389	5.8	112, 141, 243, 277, 509, 533	1..20	82
Sheep	*Ovis aries*	W5PI67	547	61,019	5.6	112, 141, 243, 277, 510, 534	1..20	82
Opossum	*Monodelphis domestica*	F7DJA1	558	63,374	5.3	129, 260, 294, 339, 457, 524, 552	1..23	75
Tasmanian devil	*Sarcophilus harrisii*	na	539	61,392	5.3	111, 140, 242, 276, 321, 505, 509, 533	1..22	74
Chicken	*Gallus gallus*	F1NFI0	601	68,047	6.6	156, 185, 287, 321, 366, 584	na	67
Lizard	*Anolis carolinensis*	H9GGQ8	524	59,239	5.8	92, 121, 223, 257, 478, 507	na	63
Frog	*Xenopus tropicalis*	A8WGX6	542	61,858	6.1	112, 141, 243, 277, 322	1..18	66
Zebra fish	*Danio rerio*	A1A5V0	561	63,771	7.7	109, 138, 181, 240, 274, 499	1..25	60
Fruit Fly	*Drosophila melanogaster*	na	502	57,760	7.3	93, 12, 22, 22, 22, 82, 60, 400	na	47

UNIPROT refers to UniprotKB/Swiss-Prot IDs for individual IDS proteins (see http://kr.expasy.org); pI refers to theoretical isoelectric points

BLAT analyses were subsequently undertaken for each of the predicted IDS amino acid sequences using the UC Santa Cruz (UCSC) Genome Browser with the default settings to obtain the predicted locations for each of the vertebrate *IDS* genes, including predicted exon boundary locations and gene sizes (Kent et al. 2002). BLAT analyses were similarly undertaken for other vertebrate *IDS* genes using previously reported sequences in each case (Table 2). Structures for human isoforms (splicing variants) were obtained using the AceView website to examine predicted gene and protein structures (Thierry-Mieg and Thierry-Mieg 2006).

**Table 2:** Vertebrate IDS Genes

*IDS* Gene	Species	RefSeq ID	GenBank ID	Chromosome location	Coding exons (strand)	Gene size (bps)
Human	*Homo sapiens*	NM_000202	BC006170	X:149,482,749–149,505,137	9 (−ve)	22,389
*IDSP1*	*Homo sapiens*	na	na	X:149,525,002–149,525,923	na	922
Chimpanzee	*Pan troglodytes*	XP_016799854	na	X:150,217,197–150,239,595	9 (−ve)	22,399
Orangutan	*Pongo abelii*	XP_002832265	na	X:149,468,629–149,491,886	9 (−ve)	23,258
Baboon	*Papio anubis*	XP_003918436	na	X:137,241,259–137,263,520	9 (−ve)	22,262
Marmoset	*Callithrix jacchus*	XP_002763402	na	X:136,661,096–136,690,421	9 (−ve)	29,326
Mouse	*Mus musculus*	NM_010498	BN000750	X:70,346,204–70,364,903	9 (−ve)	18,700
Rat	*Rattus norvegicus*	XP_017451660	BN000743	**8**:69,158,393–69,174,447	9 (−ve)	16,055
Cow	*Bos taurus*	NM_001192851	na	X:32,309,006–32,324,359	9 (−ve)	15,354
Sheep	*Ovis aries*	XP_012016345	na	X:81,295,118–81,310,976	9 (+ve)	15,859
Opossum	*Monodelphis domestica*	XP_007507328	na	X:38,769,936–38,797,831	9 (−ve)	27,896
Tasmanian devil	*Sarcophilus harrisii*	XP_012408735	na	X_GL867598:1,290,327–1,307,074	9 (−ve)	16,748
Chicken	*Gallus gallus*	XP_015133789	na	4:18,031,638–18,046,283	9 (+ve)	14,646
Lizard	*Anolis carolinensis*	XP_016851828	na	GL343310:1,066,926–1,092,995	8 (+ve)	26,070
Frog	*Xenopus tropicalis*	NM_001197132	BC154891	KB021658:33,136,298–33,145,211	9 (+ve)	8914
Zebra fish	*Danio rerio*	NM_001080068	BC128823	14:20,572,602–20,594,434	8 (−ve)	21,833
Fruit Fly	*Drosophila melanogaster*	NM_139557	AAY55004	3L:3,378,315–3,380,000	4 (+ve)	1686

GenBank IDs are derived from NCBI http://www.ncbi.nlm.nih.gov/genbank/; GL and KB refer to a scaffold; bps refers to base pairs of nucleotide sequences; the number of coding exons are listed

*RefSeq* The reference sequence, *XP* predicted sequence, *na* not available

### Predicted structures and properties of vertebrate IDS

Predicted secondary and tertiary structures for vertebrate IDS proteins were obtained using the SWISS-MODEL web-server (http://swissmodel.expasy.org/) (Schwede et al. 2003) using the reported tertiary structure for human arylsulfatase A (ARSA) (Lukatela et al. 1998; Chrusczcs et al. 2003) (PDB:1n2kA) with a modeling range of 35–549 for human IDS. Molecular weights, *N*-glycosylation sites and signal peptide cleavage sites for vertebrate IDS proteins were obtained using Expasy web tools (http://au.expasy.org/tools/pi_tool.html). The identification of conserved domains for IDS was conducted using NCBI web tools (Marchler-Bauer et al. 2011).

## Human *IDS* tissue expression

RNA-seq gene expression profiles across 53 selected tissues (or tissue segments) were examined from the public database for human *IDS*, based on expression levels for 175 individuals (GTEx Consortium 2015) (Data Source: GTEx Analysis Release V6p (dbGaP Accession phs000424.v6.p1) (http://www.gtex.org).

## Amino acid sequence alignments and phylogenetic analyses

Alignments of vertebrate and *Drosophila melanogaster* IDS sequences were undertaken using Clustal Omega, a multiple sequence alignment program (Sievers and Higgins 2014) (Table 1). Percentage identities were derived from the results of these alignments (Table 1). Phylogenetic analyses used several bioinformatic programs, coordinated using the http://www.phylogeny.fr/ bioinformatic portal, to enable alignment (MUSCLE), curation (Gblocks), phylogeny (PhyML) and tree rendering (TreeDyn), to reconstruct phylogenetic relationships (Dereeper et al. 2008). Sequences were identified as vertebrate IDS members and a proposed primordial *Drosophila melanogaster IDS* gene and protein (Tables 1, 2).

## Results and discussion

### Alignments of vertebrate IDS amino acid sequences

**Fig. 1:**
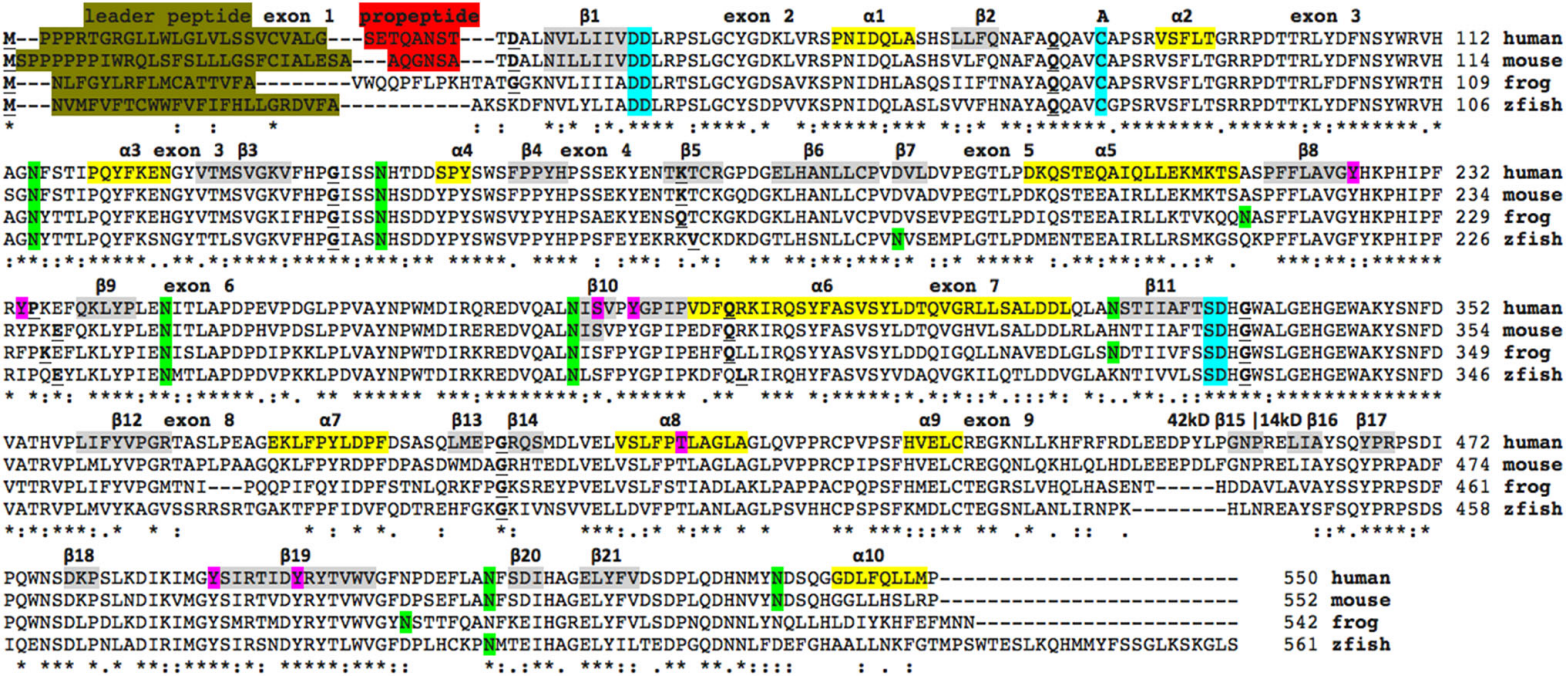
Amino acid sequence alignments for vertebrate IDS sequences. See Table 1 for sources of IDS sequences; *asterisk* shows identical residues for IDS subunits; *colon* similar alternate residues; *dot* dissimilar alternate residues; predicted phosphoresidues are in *pink*; predicted *N*-glycosylated Asn sites are in *green*; the active site residues (for human IDS) are shown in *blue*; active site residue subject to modification is shown as **A**; predicted *α*-helices for human IDS is in* shaded yellow* and numbered in sequence; predicted *β*-sheets are in *shaded gray* and also numbered in sequence from the *N*-terminus; *bold underlined font* shows residues corresponding to known or predicted exon start sites; exon numbers refer to human *IDS* gene exons; leader peptide is in *brown*; propeptide in *red*

The deduced amino acid sequences for frog (*Xenopus tropicalis*) and zebrafish (*Danio rerio*) IDS are shown in Fig. 1 together with previously reported sequences for human (Bielicki et al. 1990) and mouse IDS (Garcia et al. 2007) (Table 1). Alignments of human with other vertebrate IDS sequences examined were between 60 and 99% identical, suggesting that these are products of the same family of genes, whereas comparisons of sequence identities of vertebrate IDS proteins with other human ARS proteins exhibited ≥27% identities, indicating that these are members of distinct *ARS*-like gene families (Table 1; Supplementary Table 1).

**Fig. 2:**
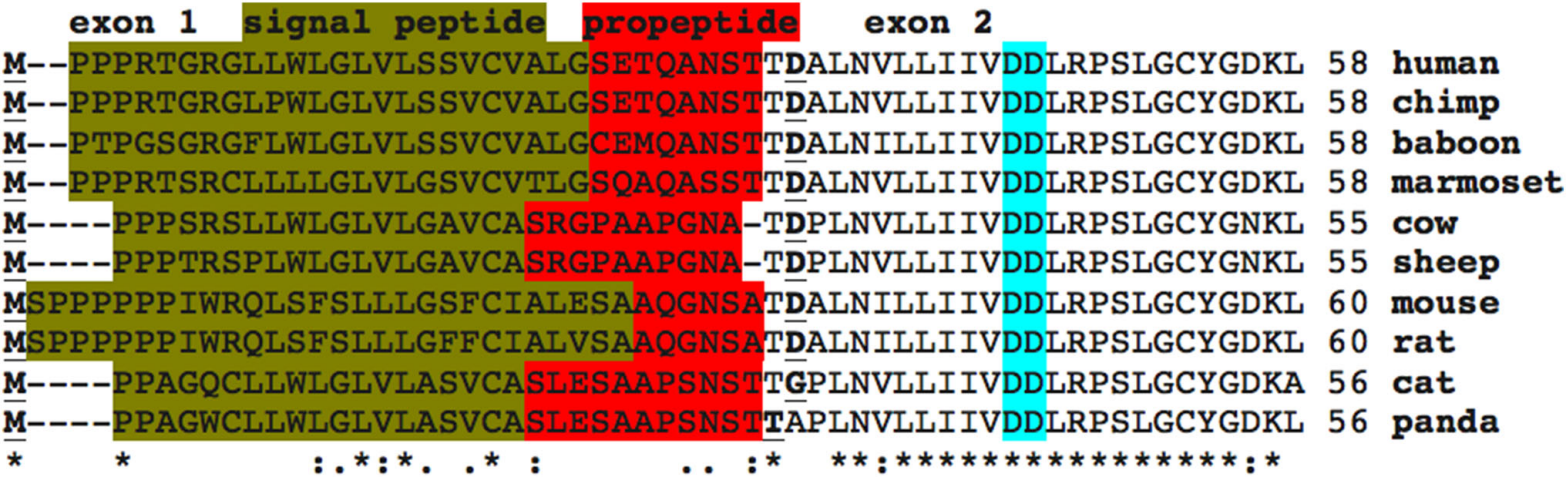
Amino acid sequence alignments for mammalian IDS *N*-terminus sequences. See Table 1 for sources of IDS sequences; *asterisk* shows identical residues for IDS subunits; *colon* similar alternate residues; *dot* dissimilar alternate residues; the active site residues (for human IDS) are shown in *blue*; leader peptide is in *brown*; propeptide in *red*; *bold underlined font* shows residues corresponding to known or predicted exon start sites; exon numbers refer to human *IDS* gene exons

The amino acid sequences for vertebrate IDS proteins contained 550–561 amino acids (Fig. 1; Table 1). Previous studies have reported several key regions and residues for human and mouse IDS proteins (human IDS amino acid residues were identified in each case) (Bielicki et al. 1990). These included an N-terminus leader peptide (24 residues excluding the *N*-terminus methionine) followed by a propeptide 8-residue segment (residues 25–33) (Wilson et al. 1990). A comparison of 10 mammalian IDS sequences for these *N*-terminal exon 1 regions revealed species specific variability in these sequences, with the signal peptides containing multiple proline and hydrophobic residues, and the propeptides exhibiting distinct mammalian sequences (see Figs. 1, 2). In contrast, amino acid sequences located further upstream within exon 2, nearer to the active site catalytic residues (Asp45; Asp46), were predominantly invariant among the mammalian and other vertebrate sequences examined (Figs. 1, 2). One of the conserved active site residues observed for these mammalian and other vertebrate IDS sequences, included an active site catalytic residue (Cys84) which undergoes post-translational modification by sulfatase modifying factor 1 (SUMF1) to form C(alpha)-formylglycine (Fgly), required at the active site of many sulfatases (Sardiello et al. 2005). Other invariant active site residues included 334Asp/335His, which are likely to be involved in Ca^2+^ binding, based on predictions derived from 3D structures from other human sulfatases (Bond et al. 1997; Hernandez-Guzman et al. 2003). An internal proteolytic cleavage has been proposed for this enzyme as a result of the presence of 42- and 14-kD polypeptides in enzyme preparations derived from human liver, kidney, lung and placenta extracts (Wilson et al. 1990) (Fig. 1). It should be noted that the 42kD polypeptide contains the N-terminal sequence with all of the active site regions, whereas the 14kD polypeptide contained the catalytically inactive C-terminus region of human IDS.

Five *N*-glycosylation sites were consistently found for vertebrate IDS sequences (human IDS amino acid sequences identified in each case): Asn115-Phe116-Ser117 (site 1); Asn144-His145-Thr173 (site 2); Asn246-Ile247-Thr248 (site 3); Asn280-Ile281-Ser282 (site 4); and Asn513-Phe514-Ser515 (site 5). Two other N-glycosylation sites were observed for human IDS which were not commonly shared with other vertebrate IDS sequences, including Asn325-Ser326-Ser327 (site 6) and Asn537-Asp538-Ser539 (site 7), the latter restricted to mammalian IDS sequences (Fig. 1; Table 1). Mutation analysis of the human *IDS* gene has shown that amino acid substitution of Asn115 (Asn→Tyr) (for site 1) resulted in Hunter’s disease, reflecting the key role of this N-glycosylation site in supporting the structure of this enzyme (Vafiadaki et al. 1998). Figure 1 also shows predicted phosphosites sites that may contribute to regulating downstream cellular processes, molecular functions and protein–protein interactions (Hornbeck et al. 2015). Five of these were strictly conserved among the vertebrate IDS sequences examined (human IDS residues: Ser282; Try285; Thr409; Tyr490; and Tyr497) supporting a role for these residues, as yet unknown.

### Predicted secondary and tertiary structures for vertebrate IDS

**Fig. 3:**
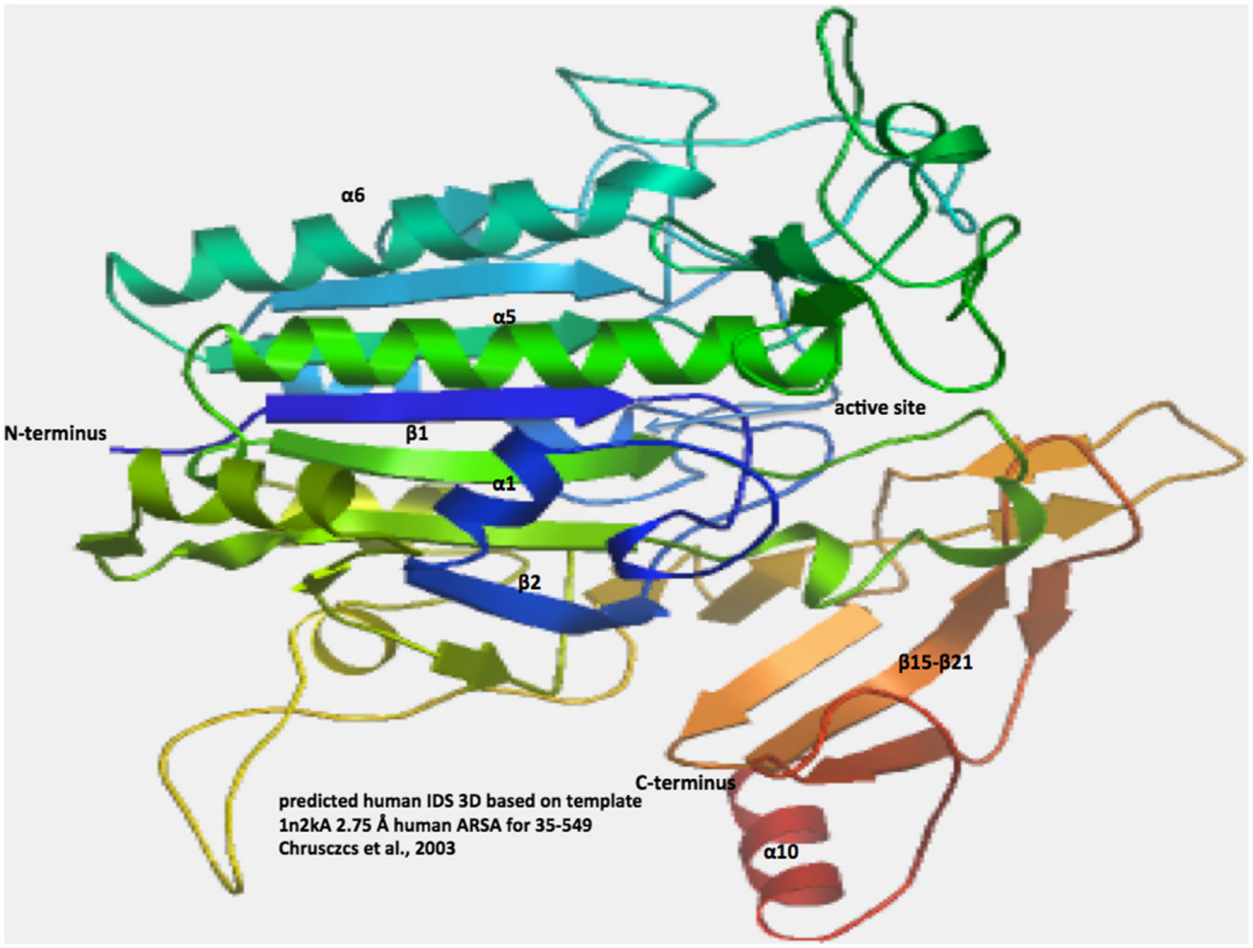
Predicted tertiary structure for human IDS. The predicted structure for human IDS is based on the reported structure for human ARSA (Chrusczcz et al. 2003) and obtained using the SWISS MODEL web site based on PDB 1N2KA http://swissmodel.expasy.org/workspace/. The *rainbow color* code describes the 3-D structures from the *N*- (*blue*) to C-termini (*red color*) for residues 35–549 for human IDS; predicted *α*-helices, *β*-sheets, proposed active site cleft, and *N*- and C-termini are shown

A predicted secondary structure for the human IDS sequence was examined (Fig. 1) using the known structure reported for human ARSA (Lukatela et al. 1998). Ten predicted α-helix and 21 β-sheet structures were observed for human IDS. Of particular interest were β-sheet structures (β1 and β11) and α-helix (α2) which were located proximate to the predicted active site residues for human IDS. The C-terminal end of human IDS contained a sequence of β-sheet structures (β15–β21), in addition to the α-helix (α10) located at the C-terminus. A predicted tertiary structure for human IDS is shown in Fig. 3. Two major domains for this enzyme were observed, that enclose a large cavity previously shown to contain the enzyme’s active site. The more N-terminal of these domains contained the active site residues and comprised the bulk of the 42kD polypeptide chain previously reported (Wilson et al. 1990), whereas the other domain comprised most of the 14kD polypeptide, including the β-sheet structures (β15–β21) and the C-terminal α-helix (α10).

### Comparative human *IDS* tissue expression

**Fig. 4:**
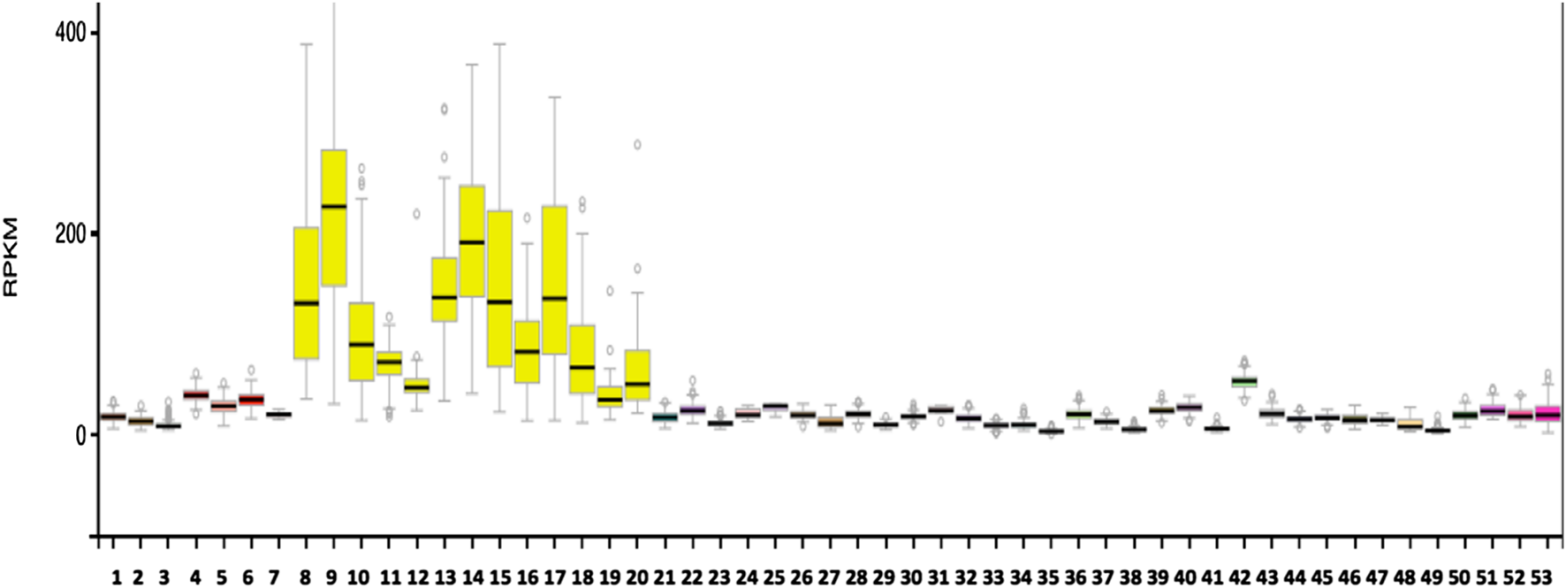
Tissue expression for human IDS. RNA-seq gene expression profiles across 53 selected tissues (or tissue segments) were examined from the public database for human *IDS*, based on expression levels for 175 individuals (Data Source: GTEx Analysis Release V6p (dbGaP Accession phs000424.v6.p1) (http://www.gtex.org). Tissues: *1*. Adipose-Subcutaneous; *2*. Adipose-Visceral (Omentum); *3*. Adrenal gland; *4*. Artery-Aorta; *5*. Artery-Coronary; *6*. Artery-Tibial; *7*. Bladder; *8*. Brain-Amygdala; *9*. Brain-Anterior cingulate Cortex (BA24); *10*. Brain-Caudate (basal ganglia); *11*. Brain-Cerebellar Hemisphere; *12*. Brain-Cerebellum; *13*. Brain-Cortex; *14*. Brain-Frontal Cortex; *15*. Brain-Hippocampus; *16*. Brain-Hypothalamus; *17*. Brain-Nucleus accumbens (basal ganglia); *18*. Brain-Putamen (basal ganglia); *19*. Brain-Spinal Cord (cervical c-1); *20*. Brain-Substantia nigra; *21*. Breast-Mammary Tissue; *22*. Cells-EBV-transformed lymphocytes; *23*. Cells-Transformed fibroblasts; *24*. Cervix-Ectocervix; *25*. Cervix-Endocervix; *26*. Colon-Sigmoid; *27*. Colon-Transverse; *28*. Esophagus-Gastroesophageal Junction; *29*. Esophagus- Mucosa; *30*. Esophagus-Muscularis; *31*. Fallopian Tube; *32*. Heart-Atrial Appendage; *33*. Heart-Left Ventricle; *34*. Kidney-Cortex; *35*. Liver; *36*. Lung; *37*. Minor Salivary Gland; *38*. Muscle-Skeletal; *39*. Nerve-Tibial; *40*. Ovary; *41*. Pancreas; *42*. Pituitary; *43*. Prostate; *44*. Skin-Not Sun Exposed (Suprapubic); *45*. Skin-Sun Exposed (Lower leg); *46*. Small Intestine-Terminal Ileum; *47*. Spleen; *48*. Stomach; *49*. Testis; *50*. Thyroid; *51*. Uterus; *52*. Vagina; *53*. Whole Blood

Figure 4 shows comparative gene expression for various human tissues obtained from RNA-seq gene expression profiles for the human *IDS* gene obtained for 53 selected tissues or tissue segments for 175 individuals (GTEx Consortium 2015) (Data Source: GTEx Analysis Release V6p (dbGaP Accession phs000424.v6.p1) (http://www.gtex.org). These data supported high levels of gene expression for human *IDS* in regions of the brain, particularly within the cortex, amygdala, hippocampus, hypothalamus and basal ganglia, but with lower levels in the brain cerebellum and spinal cord. IDS activity was also widely distributed at low levels among all other tissues examined. It is readily apparent that *IDS* is predominantly expressed in brain and nerve tissues of the body, which may reflect a specific role for IDS in neural glycoaminoglycan (GAG) metabolism, involving the efficient clearance of GAG sulfate residues within the extracellular matrix of nervous tissue.

### Gene locations, exonic structures and regulatory sequences for vertebrate *IDS* genes

Table 2 summarizes the predicted locations for vertebrate and fruit fly (*Drosophila melanogaster*) *IDS* genes based upon BLAT interrogations of several genomes using the reported sequence for human IDS (Bielicki et al. 1990; Wilson et al. 1990) and the predicted sequences for other IDS enzymes and the UCSC genome browser (Kent et al. (2002)). The predicted vertebrate *IDS* genes were transcribed on both the negative strand (primates, mouse, rat, cow, marsupial and zebra fish genomes) and the positive strand (sheep, chicken, lizard and frog genomes). Of particular interest is the X-chromosome location for *IDS* for all eutherian and marsupial mammals examined with the exception of rat *Ids* gene, which is located on an autosome (chromosome 8). This is indicative of a chromosomal transfer between the common ancestral X-chromosome and chromosome 8 during rat evolution. An *IDS* pseudogene (designated as *IDSP1*) was also observed for human and other primate genomes. Figure 1 summarizes the predicted exonic start sites for human, mouse, frog and zebra fish *IDS* genes with each having 9 coding exons, in identical or similar positions to those predicted for the human *IDS* gene. In each case, exon 1 encoded the leader peptide and propeptide with exons 2, 3 and 7 encoding the predicted active site regions for this enzyme.

**Fig. 5:**
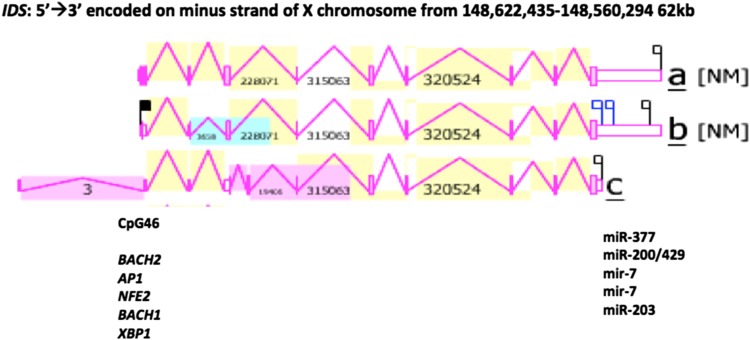
Gene structure and major gene transcripts for the human IDS gene. Derived from the AceView website http://www.ncbi.nlm.nih.gov/IEB/Research/Acembly/ (Thierry-Mieg and Thierry-Mieg 2006); shown with capped 5′- and 3′- ends for the predicted mRNA sequences; NM refers to the NCBI reference sequence; coding exons are in pink; the direction for transcription is shown as 5′ → 3′; a large CpG46 island at the gene promoter is shown (see Table 4 for details of CpG islands for human and other vertebrate *IDS* gene promoters); 5 predicted transcription factor binding sites (TFBS) for human *IDS* are shown (see Table 1s for details); 5 predicted miRNA target sites were identified within the extended 3′-UTR region of human *IDS*a and *IDSb* transcripts

Figure 5 shows the predicted structures for the three major human *IDS* transcripts (*IDSa*; *IDSb*; and *IDSc*) together with CpG46 and several transcription factor binding sites (TFBS), which are located at the 5′ end of the gene, consistent with roles in regulating the transcription of this gene and forming part of the *IDS* gene promoter. The human *IDSa* transcript was 6088 bps in length with an extended 3′-untranslated region (UTR) containing 5 microRNA target sites; the human *IDSb* transcript was 5808 bps in length, also containing 5 microRNA target sites; whereas the *IDSc* transcript was much shorter in length (2213 bps), comprising only 8 coding exons and with no microRNA target sites present. The presence of miR-200 within the 3′-UTR of the human *IDS* gene was of special interest due to this miR family being induced and having a specific role during the late stages of neuronal differentiation (Beclin et al. 2016). In addition, the presence of miR-7 in this region may also be significant given that miR-7 inhibits neuronal apoptosis in a cellular Parkinson’s disease model (Li et al. 2016) and contributes to the alteration of neuronal morphology and function (Zhang et al. 2015). Moreover, miR-203 has a proposed role as a stemness inhibitor of glioblastoma stem cells and may contribute to the increased expression of glial and neuronal differentiation markers (Deng et al. 2016).

The human *IDS* genome sequence also contained several predicted transcription factor binding sites (TFBS) and a large CpG island (CpG46) located in the 5′-untranslated promoter region of human *IDS* on the X-chromosome. CpG46 contained 432 bps with a C plus G count of 279 bps, a C or G content of 65% and showed a ratio of observed to expected CpG of 1.02. Similar CpG islands were observed in the *IDS* gene promoters for other primate, eutherian mammal, marsupial (opossum) and bird (chicken) genomes (Table 3). It is likely therefore that these *IDS* CpG islands play a key role in regulating this gene and may contribute to the very high level of gene expression observed in neural tissues (Fig. 4) (Saxanov et al. 2006). At least 5 TFBS sites were colocated with CpG46 in the human *IDS* promoter region which may contribute to the high expression of this gene in human nerve and brain tissues (Table 4). Of special interest among these transcription factor binding sites were the following: *BACH1* and *BACH2* have been recognized as members of the BTB-basic region leucine zipper transcription factor family which downregulate cell proliferation of neuroblastoma cells (Shim et al. 2006); *AP1* is constitutively upregulated in activated microglia and during the pathogenesis of Parkinson’s disease (Pal et al. 2016); *NFE2* has been shown to participate in the developmental regulation of the brain in zebrafish embryos (Williams et al. 2013); and *XBP1* has been identified as a risk factor for Alzheimer’s disease and bipolar disorders, contributing to impairment of contextual memory formation (Martinez et al. 2016).

**Table 3:** Vertebrate *IDS* CpG Islands

Vertebrate	CpG Island ID	Chromosomal position	CpG size	C count plus G count	% C or G	Ratio of observed to expected CpG
Human	CpG 46	ChrX:148,586,553–148,586,984	432	279	65	1.02
Baboon	CpG 50	ChrX:137,263,406–137,263,837	432	306	71	.92
Rhesus	CpG53	ChrX:143,222,778–143,223,221	444	318	72	.93
Mouse	CpG 26	ChrX:70,364,872–70,365,161	290	159	55	1.2
Rat	CpG 26	Chr8:69,175,527–69,175,735	209	138	66	1.14
Cow	CpG 53	ChrX:32,324,232–32,324,656	425	317	75	.9
Dog	CpG 51	ChrX;117,515,293–117,515,743	451	293	65	1.1
Opossum	CpG2 29	ChrX:38,797,675–38,797,993	319	189	59	1.05
Chicken	CpG 54	Chr4:18,031,448–18,032,009	562	333	59	1.09

The identification of *IDS* CpG islands, sequences and properties was undertaken using various vertebrate genome browsers (http://genome.ucsc.edu)

**Table 4:** Identification of transcription factor binding sites (TFBS) within the human *IDS* gene promoter

TFBS	Strand	Chr 1 Position	Function/role	Sequence	UNIPROT ID
BACH2	(+ve)	X:148,585,129–139	Binds to Maf recognition elemants	GCTGAGTCATG	Q9BYV9
AP1	(−ve)	X:148,585,128–140	Regulating cells forming the skeleton	GCATGACTCAGCT	P01101
NFE2	(+ve)	X:148,585,128–138	Regulating erythroid maturation	AGCTGAGTCAT	Q16621
BACH1	(+ve)	X:148,585,127–141	Coordinates transcription by MAFK	TAGCTGAGTCATGCA	O14867
XBP1	(+ve)	X:148,584,868–884	Regulation during ER stress	ATGGTCACATAGCCATT	P17861

The identification of TFBS within the *IDS* promoter region was undertaken using the human genome browser (http://genome.ucsc.edu); UNIPROT refers to UniprotKB/Swiss-Prot IDs for individual TFBS sequences (see http://kr.expasy.org); *ER* refers to endoplasmic reticulum

### Phylogeny and divergence of vertebrate IDS

**Fig. 6:**
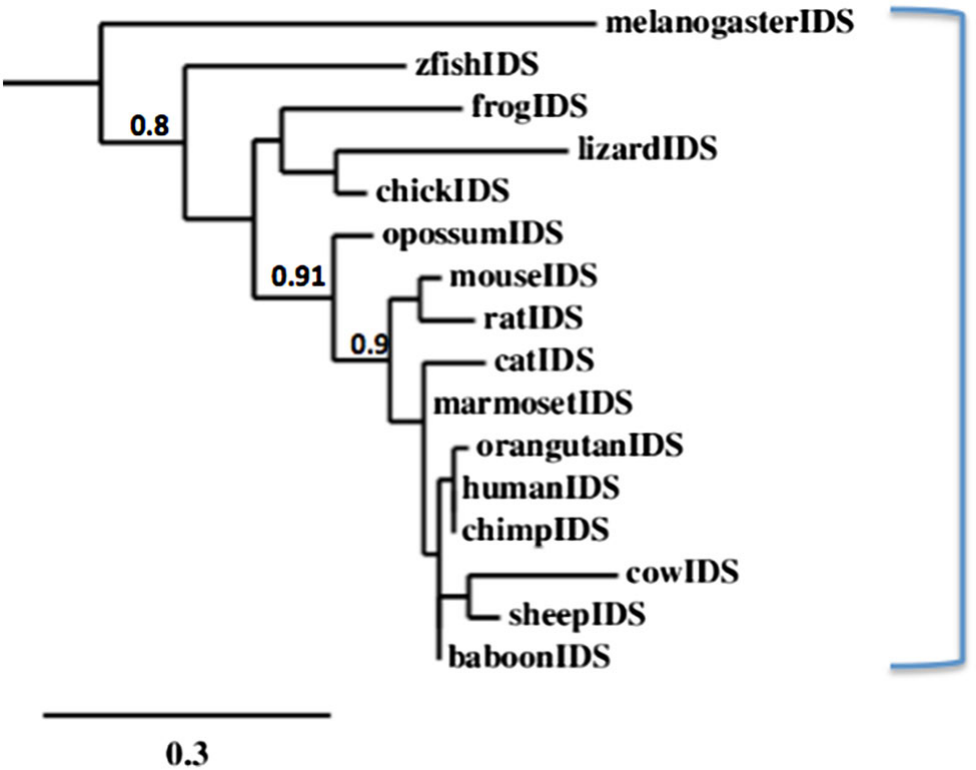
Phylogenetic tree of vertebrate IDS amino acid sequences. The tree is labeled with the vertebrate and fruit fly IDS. A genetic distance scale is shown. The number of times a clade (sequences common to a node or branch) occurred in the bootstrap replicates are shown. Replicate values of .9 or more which are highly significant (values of .9 or more), are shown with 100 bootstrap replicates performed in each case

A phylogenetic tree (Fig. 6) was calculated by the progressive alignment of 15 vertebrate IDS amino acid sequences with several other human ARS-like sequences (see Table 3). The IDS phylogram was ‘rooted’ with the fruit fly (*Drosophila melanogaster*) IDS sequence (see Table 1). The phylogram showed clustering of the IDS sequences into a single group which is represented throughout vertebrate evolution and has apparently evolved from an invertebrate *IDS* gene ancestor.

## Conclusions

The current results indicate that vertebrate *IDS* genes and encoded proteins represent a distinct gene and protein family of *ARS*-like proteins. IDS has a distinct property among human arylsulfatases in being responsible for the lysosomal degradation of the glycoaminoglycans, heparan sulfate and dermatan sulfate, by hydrolysing 2-sulfate groups of the l-iduronate 2-sulfate units (Bielicki et al. 1990). *IDS* is encoded by a single gene among the vertebrate genomes examined and is highly expressed in human brain and other nerve tissues, and contained 9 coding exons on the negative strand of the human genome. Primate genomes contained an *IDS* pseudogene (*IDSP1*) located in a proximal position on the X-chromosome. The promoter region of the human *IDS* gene contained a large CpG island together with at least 5 TFBS, which may contribute to the high level of gene expression in the brain. In addition, 5 microRNA target sites were observed within the extended 3′-UTR of the human IDS gene which may be implicated in regulating gene expression during brain development. Predicted secondary and tertiary structures for human IDS showed strong similarities with other ARS-like proteins. Several major structural domains were apparent for mammalian IDS, including the *N*-terminal leader peptide and propeptide regions; the active site (including a calcium binding site), which is responsible for arylsulfatase activity; and five conserved *N*-glycosylation sites. Phylogenetic studies using 15 vertebrate and one invertebrate (*Drosophila melanogaster*) IDS sequences indicated that the *IDS* gene has appeared early in evolution, prior to the appearance of bony fish.

## Electronic supplementary material

Below is the link to the electronic supplementary material.

Supplementary Table 1s: Percentage Amino Acid Sequence Identities for Human Arylsulfatase Proteins. Amino acid sequences for the human ARS enzymes were derived from the following: IDS (iduronate 2-sulfatase: UNIPROT: P22304); ARSA (arylsulfatase A: UNIPROT P15289); ARSB (arylsulfatase B: UNIPROT: P15848); STS (sterylsulfatase: UNIPROT P08842); ARSK (arylsulfatase K: UNIPROT: Q6UWY0); SGSH (heparan N-sulfatase: UNIPROT: P51688); SULF1 (extracellular Sulfatase 1: UNIPROT: Q8IWU6); SUMF1 (sulfatase modifying factor 1: UNIPROT: Q8NBK3). (XLSX 45 kb)

## Abbreviations

IDS: Iduronate 2-sulfatase
GAG: Glycoaminoglycan
ARS: Arylsulfatase
ARSA: Arylsulfatase A
kbps: Kilobase pairs
CpG island: Multiple C (cytosine)-G (guanine) dinucleotide region
miRNA: MicroRNA binding region
MPS: Mucopolysaccharidosis
BLAST: Basic local alignment search tool
BLAT: Blast-like alignment tool
NCBI: National Center for Biotechnology Information
SWISS-MODEL: Automated protein structure homology-modeling server
